# Effects of homophily and academic reputation in the nomination and selection of Nobel laureates

**DOI:** 10.1038/s41598-019-53657-6

**Published:** 2019-11-21

**Authors:** Riccardo Gallotti, Manlio De Domenico

**Affiliations:** CoMuNe Lab, Fondazione Bruno Kessler, Via Sommarive 18, 38123 Povo, TN Italy

**Keywords:** Human behaviour, Complex networks

## Abstract

In collective decision-making, a group of independent experts propose individual choices to reach a common decision. This is the case of competitive events such as Olympics, international Prizes or grant evaluation, where groups of experts evaluate individual performances to assign resources, e.g. scores, recognitions, or funding. However, there are systems where evaluating individual’s performance is difficult: in those cases, other factors play a relevant role, leading to unexpected emergent phenomena from micro-scale interactions. The Nobel assignment procedure, rooted on recommendations, is one of these systems. Here we unveil its network, reconstructed from official data and metadata about nominators, nominees and awardees between 1901 and 1965, consisting of almost 12,000 individuals and 17,000 nominations. We quantify the role of homophily, academic reputation of nominators and their prestige neighborhood, showing that nominees endorsed by central actors – who are part of the system’s core because of their prestigious reputation – are more likely to become laureate within a finite time scale than nominees endorsed by nominators in the periphery of the network. We propose a mechanistic model which reproduces all the salient observations and allows to design possible countermeasures to mitigate observed effects.

## Introduction

**Figure 1:**
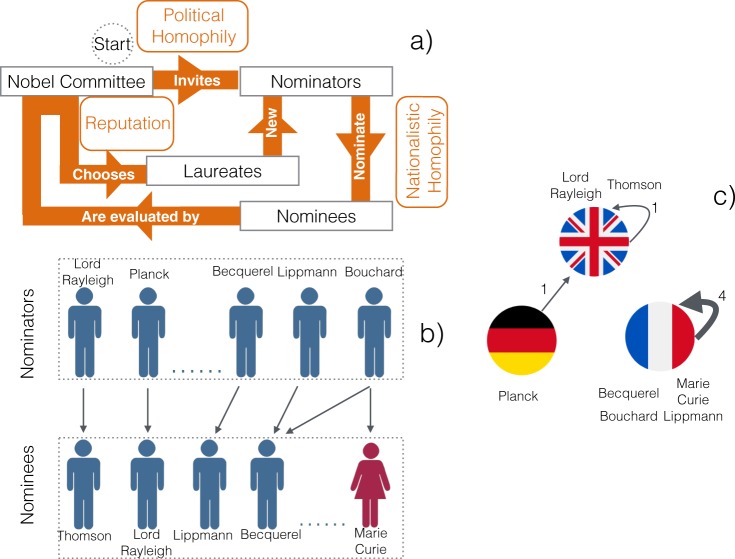
Nobel Prize assignment process. (**a**) Homophily and reputation affect different steps. In addition to those displayed, all steps are potentially influenced by gender homophily, while invitations and nominations also consider reputation and merit. (**b**) When a nominator candidates a nominee, a direct link from the former to the latter is established. (**c**) Nominators and nominees are aggregated by their country and directed links between countries are formed. Link’s weight indicates the aggregated number of nominations.

The will of the Swedish inventor Alfred Nobel established that an annual prize in his name must be awarded “to those who, during the preceding year, shall have conferred the greatest benefit to mankind” in Chemistry, Literature, Peace, Physics, and Physiology or Medicine^1^. The overall process consists of three phases (Fig. 1a): (i) nominators are invited by each category’s committee, with the exception of the Peace Prize that is open to a selected list of qualified institutional members, and include all former Nobel laureates^2^ (see also Supplementary Fig. 1); (ii) nominees for the award are proposed by competent nominators; (iii) the laureates are selected among the nominees, by Scandinavian-based committees under Nobel’s mandate that “in awarding the prizes no consideration whatever shall be given to the nationality of the candidates”. Since when the first Nobel Prizes were awarded in 1901, they quickly become globally acknowledged as the most important accolade in their fields of knowledge. In fact, winning a Nobel Prize is synonym of scientific success^3–5^ and of a globally recognized reputation^6^, that is used by academics and their institutions to quantify their prestige^7^, enhance their rank (e.g., the Shanghai Ranking^8^ scores the presence of “alumni and staff who have won Nobel Prizes and Fields Medals”) and, consequently, boosting their economic growth in a global knowledge economy^9^. At the same time, Nobel laureates also represent role models for the future generations and thus an opportunity for facilitating the vocation of sexual, gender, or color minorities in science^10^. Rapidly, the time between the publication of the awarded work and the conferment of the prize increased for all disciplines way over the single year suggested by Nobel’s will, as the awards increasingly recognised achievements that had withstood the test of time^1,11,12^. Nonetheless, the Prize has also been criticized for its winner-takes-all philosophy which has been also seen as main source of most of controversies associated with this institution^13^.

Unfortunately, quantifying academic performance is a long-standing problem, resulting in decades of research devoted to develop a wide spectrum of descriptors^14–16^. The lack of consensus on performance metrics makes it difficult to identify and rank academics, requiring alternative procedures such as recommendation networks. In fact, the academic endeavour is strongly based on social relationships of different nature^17^ and several social dynamics concur during all evaluation and assignment procedures. On the one hand, homophily is expected to be a fundamental mechanism: academics tend to better know other academics from the same institution, from the same country, or who work on the same topics. Indirectly, the numerosity of specific sub-groups (by nationality, gender, *etc*) coupled with homophily, naturally generates the hegemony of those sub-groups. On the other hand, competition for prestige and reputation is a natural mechanism^18^: academics seldom recommend direct competitors in specific fields.

Moreover, there is evidence that strong collaborations have a significant positive impact on productivity and citations^19^ – the *apostle effect* – and that author’s reputation significantly drives a paper’s citation count early in its citation life cycle^6^. Experience plays a key role in the academic community. In fact, it has been recently shown that the *chaperone effect* characterizes publishing in high-impact venues^20^, and that genealogical and coauthorship networks are good predictors of who wins multiple prizes, driving a system where the boundaries of science are pushed by small group of scientific elites^21^.

Given the scientific, economical and political impact of winning a Nobel Prize, it is natural to ask to which extent such mechanisms – namely homophily and reputation – are influenced by, and influence, the Nobel assignment process. Other factors, such as the sociological effects of winning a Nobel Prize^22^ and the patterns of productivity, collaboration, discovery, and authorship of nobel laureates have been the subject of intense research activity across half a century^5,12,23–25^ leading the emerging field of “science of science^26^”, while little attention has been dedicated to the Nobel nomination and selection mechanisms^27,28^. Here, we examine the impact of these mechanisms using the tools of network science and advanced statistics to provide compelling evidence for the emergence of four types of hegemony – political, gender, nationalistic, and prestige – influencing the three different phases of the assignment process (Fig. 1a). Similarly to other recent works on science of science^26^, our intent here is to build upon theoretical concepts and social processes drawn from the sociology of science^29,30^, isolating from large-scale data sources the power relationships present in the scientific community. This perspective is particularly relevant considering that the social structure and culture present in the scientific community influences the output of scientific knowledge produced, and that these social mechanisms can be controlled by closed circles or external forces^31^ with the undesirable effects of influencing the validity of the scientific results and overall retarding the quest for scientific knowledge by limiting the development and diffusion of new methodological or epistemological models.

To this aim, we have gathered data and metadata from the official Web page^2^ about nominators and nominees involved in the Nobel assignment procedure between 1901 and 1965, as well as about the Nobel laureates between 1901 and 2016. Both datasets have been cross-checked for inconsistencies and manually corrected where needed according to other manually curated sources, such as Wikipedia. In the data gathered, gender is indicated as a binary field – Female (F) or Male (M) – while nationality might change across time. For sake of simplicity, every person or organisation has been associated with only a single country with a majority rule.

To model the intricate web of nomination relationships, we build two networks^32,33^. One network consists of individuals, nominators and nominees, who are linked together by a nomination. For instance, Erwin Schrödinger (the nominator) nominated Erich Regener, Wolfgang Pauli and Enrico Fermi (the nominees) in 1938: in our model, three outgoing links are assigned to Schrödinger, each one pointing towards a different nominee. The second network consists of countries: a directed link is assigned to the countries to which nominator and nominees belong, with connections being weighted by the volume of nominations. For instance, a weight of one is assigned to the link from Germany (represented by Schrödinger) to Italy (represented by Fermi), whereas weight 2 is assigned to the link from United States of America to Italy, because of the nominations from Arthur H. Compton and Clinton J. Davisson to Fermi in the same year. Figure 1b,c illustrates additional examples.

To understand how homophily and reputation might affect the assignment process, we devise a model which describes (i) the progressive growth of the nomination-nominee network and (ii) the periodic assignment of an award. Our model successfully reproduces some empirical findings, such as the highly modular structures observed in the data or the central role played by prestigious scholars. Analyzing different scenarios, we illustrate how the nomination-selection process is potentially very efficient in selecting high quality laureates, but at the same time tends to perpetuate the privileges of hegemonic groups.

## Results

### Political homophily

**Figure 2:**
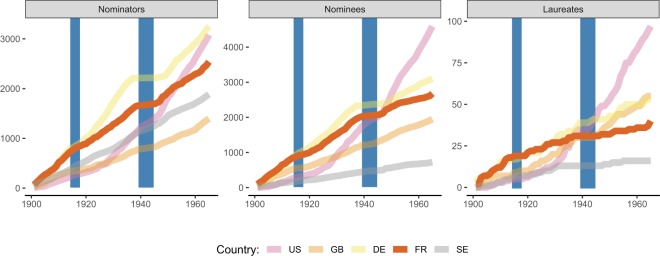
Political homophily: the effect of World World 2. The aggregated number of nominators (left), nominees (middle) and laureates (right, here a scholar is associated to the country where he/she died.) until a given year for the four most awarded countries and the “home” country of Sweden (SE). We observe clearly visible changes in trends for the number of nominators called Germany (DE) an France (FR) in correspondence of WW2 (1939–1945), which reflected in nominees and laureates. The number of laureates in particular for Germany, United Kingdom (GB) and France display a linear growth, while the USA (US) follow a super-linear growth dynamics (see also Supplementary Fig. 7 for details). A similarly accelerated growth is observed in the number of nominators and the casted corresponding candidatures but with some visible difference among prize categories (Fig. 3 and Supplementary Fig. 6). The effect of political homophily for German is confirmed by a Kolmogorov-Smirnov test on the number of candidatures in different years and categories for nominators of the different countries, separated into two intervals: 1901–1935 and 1936–1948. While for United Kingdom and Sweden we cannot reject the null hypothesis that the number of nominators is the same in the two periods, we have a significant drop of nominators for Germany (*p*–value ≈ 10^−17^) and France (*p*–value ≈ 10^−7^) and a significant growth of nominators from USA (*p*–value ≈ 10^−3^).

**Figure 3:**
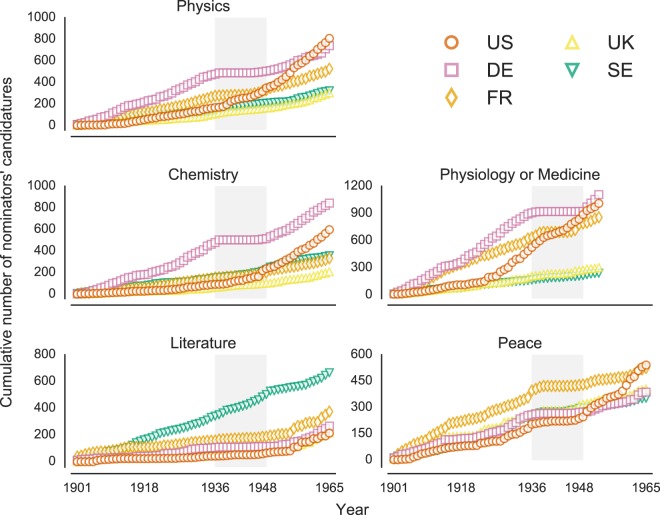
Political homophily by category. The aggregated number of candidatures casted until a given year in each category by nominators from five among the most relevant countries in the nomination process. A manifest political homophily is observed in the period between 1936 and 1948 (grey shaded area), where German scholars have been systematically excluded from the nominators pool. This growth in the US representation is characterized by a change of pace at different points in time (in the 20’s for Medicine, in the 30’s for Physics, and after the WWII for Medicine and Peace. All the trends observed are reflected by the candidatures casted (see Supplementary Fig. 6), with the exception of the observed extreme over-representation of the Prize’s home country, Sweden, in the Prize in Literature.

The rationale behind the invitation process is to broaden the representation of different countries and universities, while keeping the nominators pool restricted to qualified persons only^2^. The nominators’ selection is however influenced by international political relationships and prestige, as highlighted by the limited number of Russian nominators (114 in total, less than 10% of Americans, Germans, or French ones – see also Supplementary Fig. 2). To label this type of effect we use in this paper the term *political homophily*, which has to be intended here in the strict sense of a homophilic effect between countries sharing similar views about world politics, or economical and societal issues. Another example of political homophily is identified in correspondence of the political tensions surrounding World War II. The war indeed appear to have shocked the equilibrium of the international scientific community: if before the war the international prestige was mostly accumulated by german scholars, after the war the scientific world rewired itself into a more american-centric network (see Fig. 2 and Supplementary Fig. 3). This shock can be observed in the period between 1936^a^^1^ and 1948 for Germany, but also during the war for the German-controlled France. In these periods, nominators of these two countries have been largely excluded from the process (Figs. 2 and 3). This created a change in the nomination network before and after World War II, as the larger nominators pool taken by Germany for almost 40 years got quickly obscured by USA, which increased their weight during the war and then dominated the successive period. This naturally reflected on the nominees and laureates (Fig. 2b,c). In Fig. 3 and Supplementary Fig. 6 we disaggregate this view on the different Nobel categories, observing how in time, USA emerges with an increasing growth in both nominators and nominees pools, with the only exception of the Nobel Prize assigned to Literature. Remarkably, the overall number of American Nobel laureates grows differently from the one of all other countries (Fig. 2c). The different scaling behaviour (Supplementary Fig. 7) suggests a type of Matthew effect – also observed in other scientific contexts^34–37^ – which favors cumulative advantage of candidates with high prestige while reducing the visibility and the opportunities of less known nominees.

### Gender homophily

The Nobel Prizes assigned to women are few and far between^10^. Even after accounting for the underlying under-representation of women in the scientific disciplines, the assignment of Nobel Prizes is significantly favouring men^38^. In the period we consider (1901–1965) we restricted the analysis to the 15668 nominations where both the gender of the nominators and of the nominees was correctly identified. In this sample, women constitute 5.0% of nominees and 3.7% of laureates but only 1.8% of nominators, highlighting natural limitations for women to enter in the nominators’ pool. To investigate the role played by gender homophily in the nomination process, we count the fraction of links between nodes of the same (F → F and M → M) or different genders (F → M and M → F) and compare them with a null model where the gender is randomly shuffled among the nodes. The results, displayed in Table 1, show without any doubt^b^^2^ significant effects of gender homophily the nomination process (Z-Score ≈ 13). The effect is symmetrical, as both genders equally favour intra-gender links with respect to the null-model.

**Table 1:** Intra- and inter- gender links in the nominator-nominee network.

Link Type	Data	Null Model	Z-Score
F → F	56	11.6 ± 3	13.5
F → M	173	217 ± 3	−13.5
M → F	740	784 ± 3	−13.2
M → M	14699	14655 ± 3	13.2

We compare the data with the result of 100000 random shuffling of the nodes genders. The observed deviations are of the order of ≈13 standard deviations, which essentially correspond to certainty.

### Nationalistic homophily

**Figure 4:**
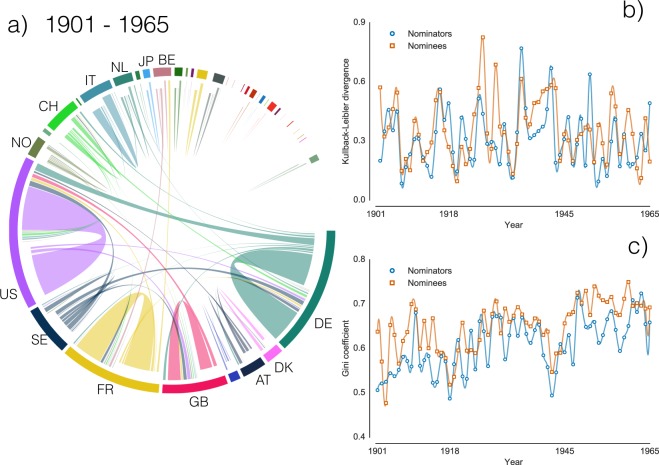
Nationalistic homophily. (**a**) Nominators and nominees build an intricate web of relationships. By aggregating the number of nominations among countries across time, we show their directionality and their overall volume between 1901 and 1965 with a circular diagram^50^. (**b**) The statistical difference between the distributions of countries in two following years, quantified by the Kullback-Liebler divergence (see Methods), is calculated for nominators and nominees across time. The two trends are highly correlated (Spearman index 0.47, *p*–value ≈ 2.1 × 10^−5^). (**c**) Diversity of nominators’ and nominees’ origin country for a given year quantified by the Gini coefficient (see Methods): the higher the value the more unequal is the distribution of nominators or nominees among the different countries. The coefficient is calculated across time and it exhibits a growing trend, which is highly correlated (Spearman index 0.68, *p*–value ≈ 10^−9^) with the trend of the Gini coefficient for nominators. Note the significant drop corresponding to WWII.

Through their nominations, recognized experts propose and support the candidate who, in their opinion, deserves the most the Nobel Prize. However, candidatures are more likely towards fellow academics from the same country. To quantify this effect during the nomination process, we use the network of nominations at country level, aggregated across time. Our analysis reveals a large fraction of nominations among individuals from the same country: the level of clustering into communities is quantified by network modularity^39^ – calculated with respect to a country-based partition – for which a value of 0.38 is measured. Considering each Nobel category separately, the highest modularity (0.44) is observed for Physiology and Medicine and for Literature, while lowest values characterize Chemistry (0.34), Peace (0.32), and Physics (0.28). These high values indicate that the fraction of nominations within the same country exceeds what would be expected by chance, highlighting the existence of a nationalistic homophily, which appears to depend on the historical moment (see Fig. 4a, and Supplementary Figs. 3 and 4 for a comparison before and after World War II). This type of homophily reflects in the country distribution of the nominees, which is therefore strongly related to the committee choices of nominators. To verify this claim, we measure the evolution of the nominator and nominees pool countries with the Kullback-Liebler divergence (see Methods) between the distribution of countries in two consecutive years. The results shown in Fig. 4b confirm our expectation as the yearly evolution of the nominators and nominees pools is significantly correlated (Spearman *r* = 0.47). Consequently, as the nominators pool gets progressively concentrated in a few countries, the nationalistic homophily propagates this concentration to the nominees. Indeed, measuring the statistical dispersion of both distributions across time by means of the Gini coefficient (see Methods), a widely adopted index of diversity^40^, we observe how the candidatures increasingly concentrate in fewer countries (Fig. 4c) regardless of the Nobel category (Supplementary Fig. 8). The trend is the same for both nominators and nominees, and the two dynamics exhibit a highly significant correlation (Spearman *r* = 0.68). These results show that the pool of nominees strongly depends on the nominator pool, a fact that contributes to dramatically alter the probability that a nominee will become a Nobel laureate.

### Academic reputation

The committees, supported by specially appointed experts, choose the laureates among the nominees, in a process influenced by the committee members expertise and preferences^27^. The number of nominations, on average five for awardees and two for non-awardees, is likely to play a role in the process. However, we have isolated an important effect due to prestige: the Nobel committee attributes greater accuracy to the opinion of former Nobel laureates, and it is particularly important if the initial candidature is endorsed by former Nobel laureates.

**Figure 5:**
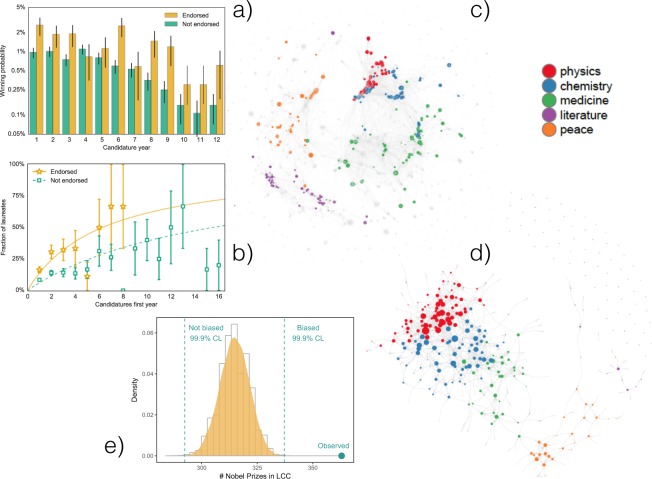
Academic reputation and prestige. (**a**) Probability over time that nominees “Endorsed” by a former Nobel laureate in their first year of candidature get awarded. Probability for all other cases (“Not endorsed”) is also shown. The difference is significant, the endorsement representing, on average, a probability 250% higher to get awarded. (**b**) The chances of being endorsed is larger if one has many nominations in the first year. However, endorsed are advantaged over not-endorsed even when the number of nominations is the same. Not-endorsed nominees need, on average, 2.75 times more nominations in their first year to achieve the same winning rate as the endorsed ones. (**c**) Nomination network. Nodes are nominators and nominees, colors highlight Nobel laureates in different disciplines. (**d**) Nobel laureates build a strongly interconnected core. A scientific elite is identified, with laureates in Literature and Peace forming a cloud surrounding the core. (**e**) 363 Nobel laureates are observed in the largest connected component of the nomination network. Under the null hypothesis of an unbiased process, this number is 7.1 standard deviations larger than expectation.

Such candidatures are indeed dramatically advantaged with respect to those ones not initially endorsed by Nobel laureates (Fig. 5a,b and Supplementary Figs. 9 and 10). To further test the hypothesis that the observed effect is genuine, we studied the nomination network (Fig. 5c) at individual level, to gain insight from the microscopic analysis of the Nobel assignment system. We find that Nobel laureates in Physics, Chemistry and Medicine are part of a scientific elite (Fig. 5d), constituting the system’s core and counting 363 individuals in the largest connected component of the nomination network. To quantify the chance of this observation, we have reshuffled the Nobel Prize assignments 50,000 times and counted, each time, the number of Nobel laureates in the largest cluster^32,33^. The random expectation, compatible with the null hypothesis that the endorsement of former Nobel laureates is not a discriminating factor, is 314.5 ± 6.8: remarkably, the empirical value is more than 7 standard deviations from the mean (*p*–value ≈ 10^−12^), confirming the significant presence of a core (Fig. 5e). The authority of Nobel laureates thus induces a sort of social influence that is reflected in the importance given by the Nobel committee to their nominations, thus affecting the collective judgement^41^.

Similarly, another indicator of academic prestige we identified is having being repeatedly selected as a nominator. In Supplementary Fig. 10 we show how nominators who casted more candidatures are more likely to nominate winning candidates.

### A mechanistic model of the Nobel ecosystem

**Figure 6:**
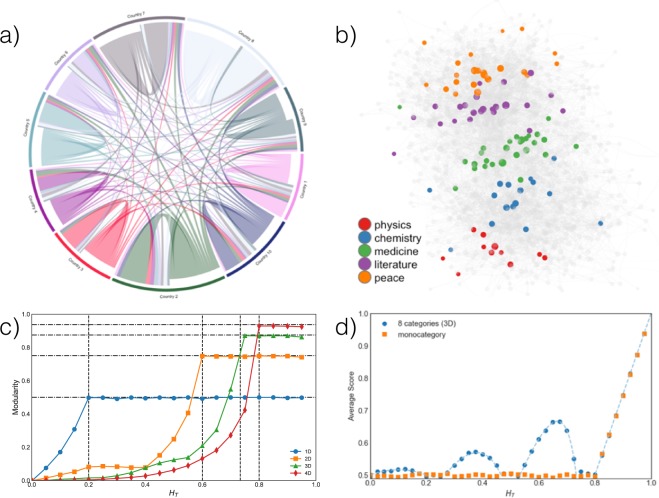
Network model. Using different feature spaces allows us to describe different inter-group structures. (**a**) Nationalistic modularity. A feature space constituted by ten 10-dimensional feature vectors ((+1, −1, −1, −1, −1, −1, −1, −1, −1, −1), (−1, +1, −1, −1, −1, −1, −1, −1, −1, −1), …) allows for describing the relationships between countries. The example here represented has been generated with *B* = 0.2, *H*_*T*_ = 0.82 and assumes countries of identical size. Similarly to Fig. 4a, we have here a high modularity *Q* = 0.4. (**b**) Nobel categories. Using five 3-dimensional feature vectors ((+1, −1, −1), (0, 0, −1), (−1, +1, −1), (−1, 0, 0), (−1, −1, +1)) we can describe the relation between Nobel categories. This example is generated with *B* = 0.2, *H*_*T*_ = 0.6, and having modularity *Q* = 0.2. Similarly to Fig. 5c,d, color highlights laureates in the different disciplines, that are arranged in clear bands. (**c**) Non overlapping communities. Simulating numerically the evolution for the model with random features uniformly distributed in a N-dimensional cubic feature space (e.g. 2D: (+1.+1) (+1, −1) (−1, +1) (−1, −1)) we can produce highly modular structures. The values of modularity reaches a maximum value Q*(*D*) (dot-dash line) for *H*_*T*_ over a threshold value ${H}_{T}^{\ast }(D)$ (dash lines). Both values depend on the dimensionality of the feature space (see Supplementary Information). (**d**) Better selection of dissimilar nodes. The presence of categories allows for a more selective choice of “diverse” nodes. We show here the comparison between the average score of candidate nodes in a system where all 8 possible categories of a 3D cubic feature space are allowed, against the average score when only a single category is present. The simulations are run with *B* = 0.2, for which nodes with *S* < 1 can be connected only if all nodes with *S* = 1 are already. If only a single category is allowed (orange squares), nodes are selected according to their score only if *H*_*T*_ > 0.8, and below such value all nodes are accepted for nomination and 〈*s*〉 = 0.5. If all categories are present, under the same conditions the selection process is able to filter better nodes among the more distant categories (blue circles). See Methods for more details. All simulations in this figure are made with random values of *s* uniformly distributed in [0, 1], *N*_0_ = 15, *N* = 1000, *L* = 10, *α* = 0 (no aging), *M* = 0 (no laureates injected in the nominators pool).

To better understand our empirical findings, we develop a model describing the Nobel ecosystem, including the growth of its nomination network. In this network, nodes represent experts that can be at the same time both nominators and nominees. Links between nodes are directed and indicate a nomination. Each node *i* is characterized by (i) a random score *s*_*i*_, distributed uniformly between 0 and 1, which embodies the individual expertise and merit; (ii) the node age *a*_*i*_ measured in time-steps; and iii) a vector of features ${\overrightarrow{F}}_{i}$ ^42^ encoding information such as nationality, gender, *etc*. The network is first initialized with *N*_0_ nodes and no links: these initial nodes represent a starting core of nominators, that are never considered as potential nominees. Then, at every time step, a new node is injected, and a set of *L* potential nominators is selected on the base of their score and, eventually, their age with probability ${p}_{i}^{out}=\frac{{a}_{i}^{\alpha }{s}_{i}}{{\sum }_{i}\,{a}_{i}^{\alpha }{s}_{i}}$. Here, the score is multiplied by an aging factor ${a}_{i}^{\alpha }$. If *α* > 0, it is representing the social advantage cumulated along the career^43^, while if *α* < 0 young nodes are favoured in the selection. If *α* = 0, age has no weight in the choice. These *L* potential nominators might then connect or not with the new node, the choice corresponding to deciding on whether to support or not the candidature of the new node. The evaluation of a node is based, simultaneously, upon reputation (score) and homophilic tendencies (defined in the feature space). Therefore, it is crucial to define the similarity $S({\overrightarrow{F}}_{1},{\overrightarrow{F}}_{2})$ between two nodes’ feature vectors. Here, we define similarity as $S({\overrightarrow{F}}_{1},{\overrightarrow{F}}_{2})=(1+\,\cos \,sim\,({\overrightarrow{F}}_{1},{\overrightarrow{F}}_{2}))/2$ where

1 $$cossim\,({\overrightarrow{F}}_{1},{\overrightarrow{F}}_{2})=\frac{{\overrightarrow{F}}_{1}\cdot {\overrightarrow{F}}_{2}}{\Vert {\overrightarrow{F}}_{1}\Vert \Vert {\overrightarrow{F}}_{2}\Vert }$$

is the cosine similarity. *S* = 1 if the features are parallel, *S* = 0 if they are orthogonal. This choice differs for example to the more commonly used Axelrod’s model^44^, where the similarity is given by the fraction of shared features, and has the advantage that the cosine similarity induces an ordering among the feature vectors. Similarly to bounded confidence models^45^, interactions (here, the nominations) are possible only if the nodes are not too different. However, here differences can be ignored if the reputation is high enough. The possible new link between *i*_1_ and *i*_2_ is indeed accepted, deterministically, only if

2 $$B{s}_{{i}_{2}}+(1-B)S({\overrightarrow{F}}_{{i}_{1}},{\overrightarrow{F}}_{{i}_{2}})\ge {H}_{T},$$

where *B* is a parameter describing the *meritocracy* of the choice (if *B* = 1, the choice is purely based on score), *H*_*T*_ a threshold parameter, This framework allows one to design block-like adjacency matrices describing the complex community structures observed in our data, including the case groups’in-between’ other two (Fig. 6a,b). For instance, in our particular problem – the study of the Nobel Prize nomination process – the added value of this perspective is manifest as it allow us to map the fluid relationships between scientific disciplines^25^ (as illustrated for example in Fig. 6b). Similarly to what observed for the Axelrod’s model^46^, one possible output here is the creation of segregated non-overlapping communities, consisting of nodes of identical features (See Fig. 6d, Supplementary Fig. 11 and the Supplementary Information).

The fact that nodes’ scores are considered for both the nominators selection and the nomination naturally segregates a high score core from a low score periphery. This property reflects what we observed in the data with the high concentration of Nobel laureates in the core. Another remarkable consequence of the homophilic link creation is that, in certain regimes, the presence of different categories of nodes makes the average score of selected nodes higher than what observed *ceteris paribus* when all nodes injected belong to the same category (see Fig. 6e). In a nutshell (see Methods for more detail), the process allows for selecting the best nodes among distant categories, even when the meritocracy is relatively small and closer categories are accepted without any regard of node score.

However, together with this relatively positive consequence, the presence of nodes of different categories might naturally yield also some negative effect. One example worth highlighting here is the persistent influence that a hegemonic group may play if the selection process is driven by a strong memory effect, as is the case in the Nobel nomination network. To illustrate this effect, we introduce in the model two further features inspired by the Nobel selection process. First, every *T* time-steps, a prize is awarded with a probability ${p}_{i}^{award}=\frac{{k}_{i}}{{\sum }_{j\in J}\,{k}_{j}}$ proportional to the node in-degree *k*_*i*_. This selection is restricted to the set *J* of nodes that are not yet laureate (multiple awards are not permitted). Second, besides the *L* nominators selected accordingly to skill and, eventually, age, also the last *M* laureates are included in the nominators pool, and are similarly allowed to decide whether or not to nominate a new node with the outgoing directed link representing. This ‘design choice’ – of making Nobel laureates systematically become nominators – strengthens the central position of skilled nominators at the core of the network. In the following, we show that it has the drawback of perpetuating for longer time the influence of a hegemonic initial pool of nominators, established as the initial set of *N*_0_ nodes.

**Figure 7:**
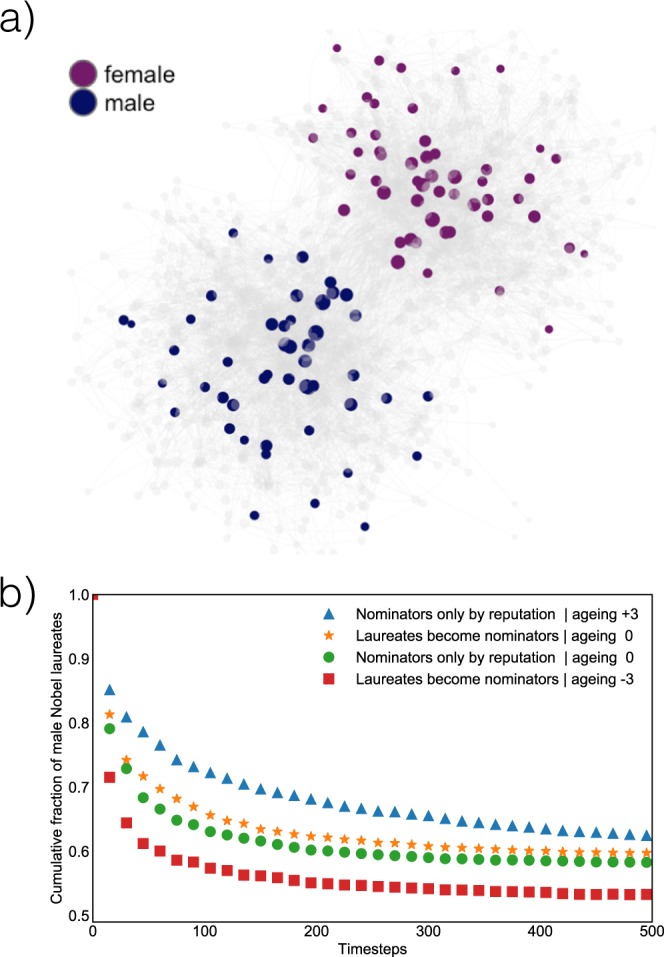
Modeling the persistence of a gender hegemony. (**a**) Gender-modular network. With a simple mono-dimensional feature space ((+1), (−1)) we can describe a nomination network with high gender-modular (*Q* = 0.4, with *H*_*T*_ = 0.18, *B* = 0.2, all other parameters as described in Fig. 6). (**b**) Persistence of an initial hegemony. On a network generated as in panel (a), all initial *N*_0_ nominators are set to be males. The process lasts for 500 timesteps, and every 10 timesteps a Prize is awarded. We study four different scenarios average over 200 simulations each. In all cases the curve begins from 1 and progressively drops as the initial hegemony is challenged. Note that, unless for a period the nominators becomes unbalanced in the other sense, the fraction will be strictly >0.5 as the initial gender gap cannot be recovered in a fair system. The drop in hegemonic weight is slower with positive aging and faster with negative aging. The inclusion of former laureates in the nominator pool beside other normally selected nodes (with *L* = *M* = 10) significantly increase the memory effect, actively sustaining the initial hegemony.

To illustrate this, we study the simple scenario of a mono-dimensional feature space with only two possible types of nodes: (+1) and (−1). The two-features scenario allows only for identical *S* = 1 or orthogonal *S* = 0 pairs. To simplify the interpretation of the results, let us assume that this scenario describe gender homophilic decisions in the Nobel Prize. First, we can analyze the model without the new feature imposing the last former laureates as nominees, and with non-hegemonic initial conditions. We show in Fig. 7a an example of a network in this first non-hegemonic scenario, with modularity ≈0.4 built with *B* = 0.2 and *H*_*T*_ = 0.18.

For this example, in Fig. 7b we measure the gender unbalance in four different scenarios as the cumulative fraction of the number of awards assigned among the hegemonic gender until a given time. The baseline scenario is represented by the orange green circles, where there are no aging effects and the mechanics of injecting the laureates as nominators is not activated. This has to be compared with three variated scenarios. The positive aging scenario (blue triangles) introduces an extra age effect to reputation, describing a system where further social advantage is cumulated along the career. In this case the system has a stronger memory and the hegemony is sustained for longer times. The realistic scenario (orange stars) is without aging, but here former laureates automatically become nominators, as is the case for the Nobel prize. Similarly to the positive aging scenario, the system has a stronger memory and the hegemonic initial unbalance is maintained for longer times. The last (red square) is the negative aging scenario. It is similar to the realistic one, but here a negative aging parameter favours youngest nodes as nominators. In this last case, the memory is reduced and the effect due to hegemonic initial condition and high homophily is limited. This example suggests that the current design, where Nobel laureates are automatically included as nominators, creates a memory effect that might perpetuate existing hegemonies. The same result is found for a very broad range of the parameters *B* and *H*_*T*_ (see Supplementary Fig. 12).

## Discussion

Interestingly, starting 2019 the Nobel committees explicitly request the nominators of considering diversity in geography, gender, and topic^10,47^. Further measures have also been requested to improve gender balance, including changes in the nomination committee and nomination rules^47^. Here, we have shown that these requests are definitively justified. Nominations are also surely gender biased, with both females and males preferring candidates of the same gender. The winning odds would be however fair if the choice of nominators would be gender balanced in the first place, but this was definitively not the case in the period 1901–1965, where official data and metadata are publicly available.

There is also evidence that nominations are mostly affected by nationalistic homophily. The nomination network is highly modular with respect to the country of origin, *de facto* making more difficult to award candidates from less represented countries, further increasing inequality. This effect can be originated by different mechanisms – e.g. nominators’ limited social/scientific neighborhood or real nationalistic preferences – whose determination is, however, beyond the scope of this work. Nevertheless, the existence of this type of homophily – similar to the one discovered in other highly competitive events, such as Olympics^48^, and in social dynamics^49^ – represents a huge obstacle to the fairness of the overall assignment process. The sum of all these effects renders the ultimate decision of who, among the candidate available, will win the Nobel prize highly predictable from the aggregated history of nominations up to that year. An additional evidence to support this argument is given by a machine learning algorithm able to learn these patterns, as we show in Supplementary Fig. 13.

This process is further influenced by political homophily and perceived academic prestige in the committees. Our results indicate that the Nobel assignment procedure (see Fig. 1a) is intrinsically reinforcing the propagation of these homophilic effects sustaining the presence of academic hegemonies over time. In particular, these effects are aggravated by the current prize assignment mechanism allowing new Nobel laureates to become nominators in the subsequent years, an undesired effect that can be reduced by selecting new young experts as nominators, as suggested by our model.

More in general, having pointed out a number of social mechanisms that influence the Nobel selection process, a natural question that arises is about the relative strength of such mechanisms and where eventually one may intervene to reduce the biases emerging from these. In this sense, we are inclined to conjecture that the single most efficient intervention would be to have the Nobel committees unbiased in terms of the homophilic tendencies highlighted in this paper. Homophily would be less an issue if the committee would not display hegemonic prevalences in terms of nationality and gender.

## Materials and Methods

### Kullback-Leibler divergence

The Kullback-Leibler divergence is a measure of “surprise”, quantifying how much a distribution *P*(*x*) can be well described by another distribution *Q*(*x*), where *x* is some observable of interest. Formally, it is defined by

$${D}_{{\rm{KL}}}(P||Q)={\int }_{-\infty }^{\infty }P(x)\,{\log }_{2}\frac{P(x)}{Q(x)}dx$$

quantifying information loss in describing *P*(*x*) by means of *Q*(*x*). A divergence close to zero indicates that the two distribution are very similar, if not identical. Conversely, larger the difference between the two distributions, larger the expected value of their divergence. In this work, we consider the distribution of the countries of nominators and nominees, separately, and we calculate their Kullback-Leibler divergence between successive years to quantify the underlying similarity across time.

### Gini coefficient

The Gini coefficient is a measure of statistical dispersion, originally introduced to quantify income and wealth inequality. Formally, it is derived from the Lorenz curve *L*_*P*_(*y*) of the probability distribution *P*(*x*), which describes the relative weight of the bottom *y*% items of the sample from *P*(*x*), as

$$G(P)=2{\int }_{0}^{100 \% }(y-L(y))dy$$

and thus represents the relative dimension of the inequality gap between the line of perfect equality and the Lorenz curve observed for the distribution at hand. The coefficient ranges from 0 to 1. A Gini coefficient of 0 represents perfect equality, while maximal inequality among the recorded values corresponds to a value of 1. Intermediate values, such as 0.5, characterize, for instance, a relatively high income inequality for a country. In this work, we measure the Gini coefficient of the distribution of the countries of nominators and nominees for a given year.

### Computing the winning probabilities

To isolate the academic reputation, we have studied in Fig. 5 and Supplementary Fig. 9 the sequences of candidature years for different nominees. In this analysis, years are not necessarily consecutive. Moreover, in Fig. 5a the sequences representing the years of candidatures of non-laureated nominees that were shorter than 12 items have been extended to that length, as being excluded from the nomination process implies the impossibility of being awarded.

### Selection with multiple groups

We observed in Fig. 6e that the model proposed is better at selecting high score nodes if the system is equally constituted by multiple categories. This apparently counterintuitive effect can be easily understood by noticing that, by definition, the similarity within the same category is *S*_1_ = 1, while an eventual second closest category has a similarity *S*_2_ = 1 − Δ*S*. For sake of simplicity, let us consider the case *B* < Δ*S*/(1 + Δ*S*) where nodes of the second category can be selected only for values of *H*_*T*_ ≤ (1 − *B*). This last condition corresponds to requiring that all nodes of the first category are automatically accepted, and consequently that the average score for the nodes of the same category of 〈*s*_1_〉 = 0.5. Since *s* is distributed uniformly between 0 and 1, the values of *H*_*T*_ act as a cursor selecting a fraction *f*_2_ ∈ [0, 1] of nodes that pass a threshold *H*_*T*_ = (1 − *f*_2_)*B* + (1 − *B*)(1 − Δ*S*). These nodes are those with the highest scores among the second category will average 〈*s*_2_〉 = 1 − *f*_2_/2 > 〈*s*_1_〉. In total, the average score for any accepted node with *H*_*T*_ activating links in the first and second categories is given by the weighted average 〈*s*〉 = (*n*_1_〈*s*_1_〉 + *n*_2_*f*_2_〈*s*_2_〉)/(*n*_1_ + *n*_2_*f*_2_) > 〈*s*_1_〉, where *n*_1_ is the fraction of nodes with similarity *S* = 1 with a randomly chosen node, and *n*_2_ the fraction of nodes with similarity *S*_2_ with a random node (see Fig. 6d, where the dashed line indicates the analytical solution found with the principles described here above). This last inequality states that the average score 〈*s*〉 exceeds the averages score 〈*s*_1_〉 one will have if all nodes belong to the same category.

## Supplementary information

Supplementary Information

## Data Availability

Data are available from the authors upon request.

## Footnotes

1 Year following the promulgation of the antisemitic and racist laws in Nazi Germany.

2 We verified that the distribution produced by the null model is homogeneous (see Supplementary Fig. 5), and statistically compatible with a *p*–value of 0. For sake of completeness, the most conservative statistical test – given by the Chebyshev inequality – suggests that this observation is likely with probability 5.7 · 10^−3^.
