# Supplementary material for: Effects of homophily and academic reputation in the nomination and selection of Nobel laureates
Source: Sci Rep. 2019 Nov 21;9:17304. doi: 10.1038/s41598-019-53657-6 (PMC6872660; doi:10.1038/s41598-019-53657-6)
Supplement: Supplementary file 1 — Supplementary Information [file 41598_2019_53657_MOESM1_ESM.pdf]

Supplementary Information for: Effects of homophily and academic reputation in the nomination and selection of Nobel laureates

Riccardo Gallotti[1] and Manlio De Domenico[1*]

[1]Fondazione Bruno Kessler, Via Sommarive 18, 38123 Povo (TN), Italy.
[*]To whom correspondence should be addressed; E-mail: mdedomenico@fbk.eu.

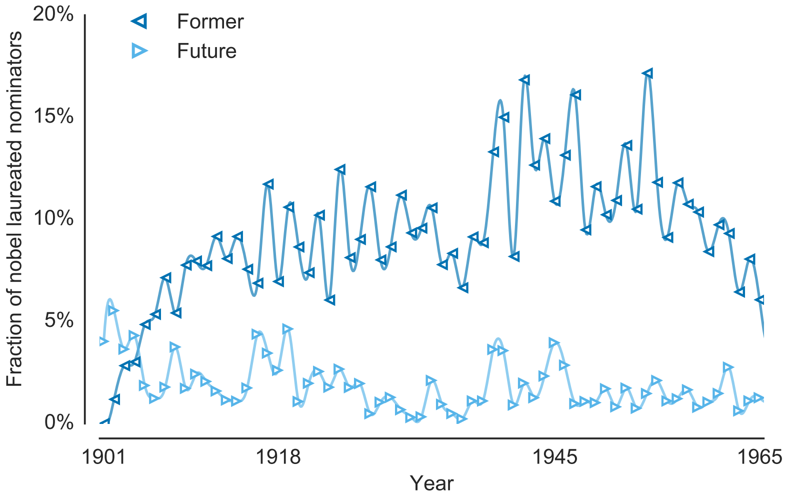

Supplementary Figure 1. Fraction of Nobel laureates (former or future) among the nominators. Some nominators are already Nobel laureates at the time when their nominations are casted ("Former"), while a smaller fraction of nominators will the receive the award in the future ("Future"). The "Former" Nobel laureates progressively took a significant share ($\approx 10\%$) of the nominator pool, while "Future" laureates represent a smaller share ($\approx 2\%$).

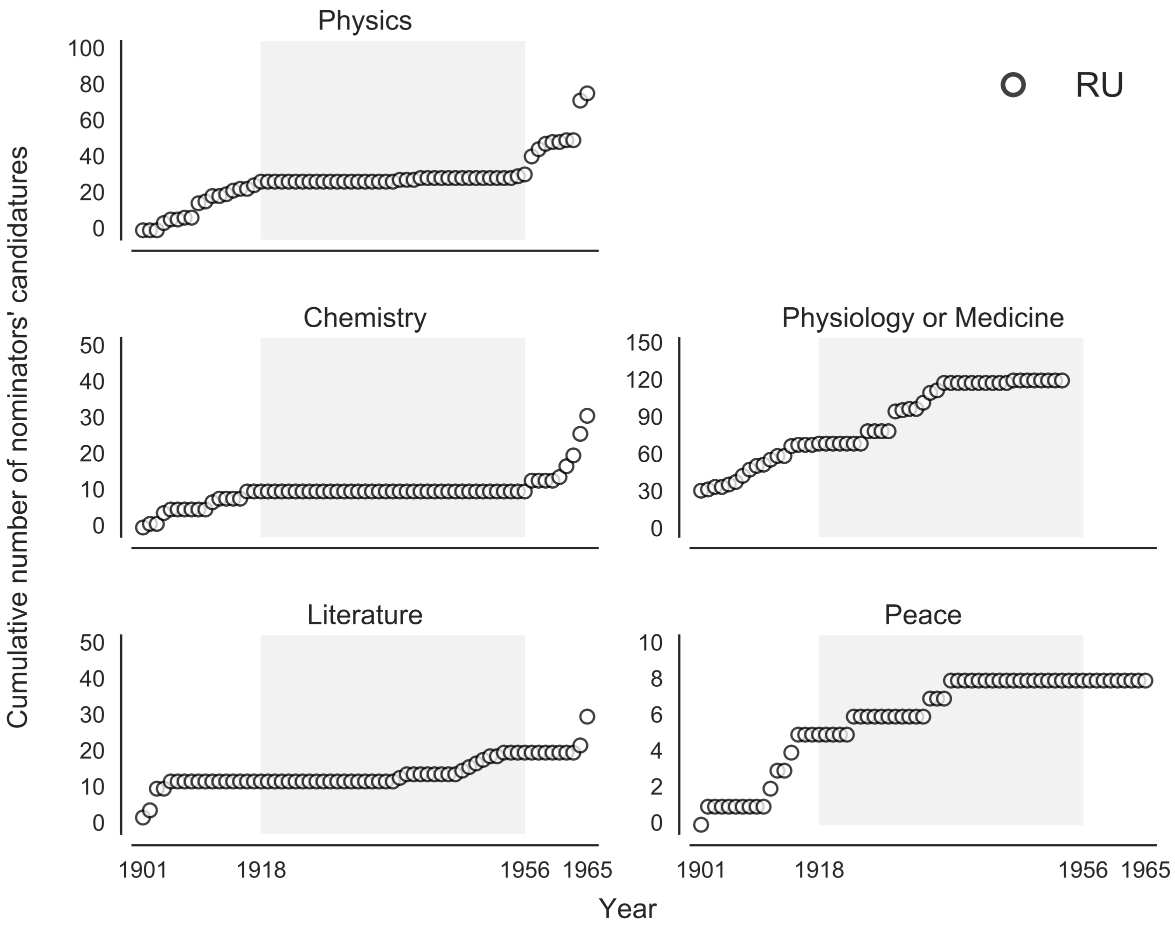

Supplementary Figure 2. Political bias with the Soviet Union. Similarly to Fig. 3, we show the aggregated number of candidatures casted until a given year by Russian nominators in the different categories. The trend here changes among the five categories. For Physics and Chemistry a political bias can be observed in the period between 1918 and 1956 (grey shaded area), suggesting that Soviet scientists have been systematically excluded from the nomination process until the de-Stalinization happened in the Khrushchev era.

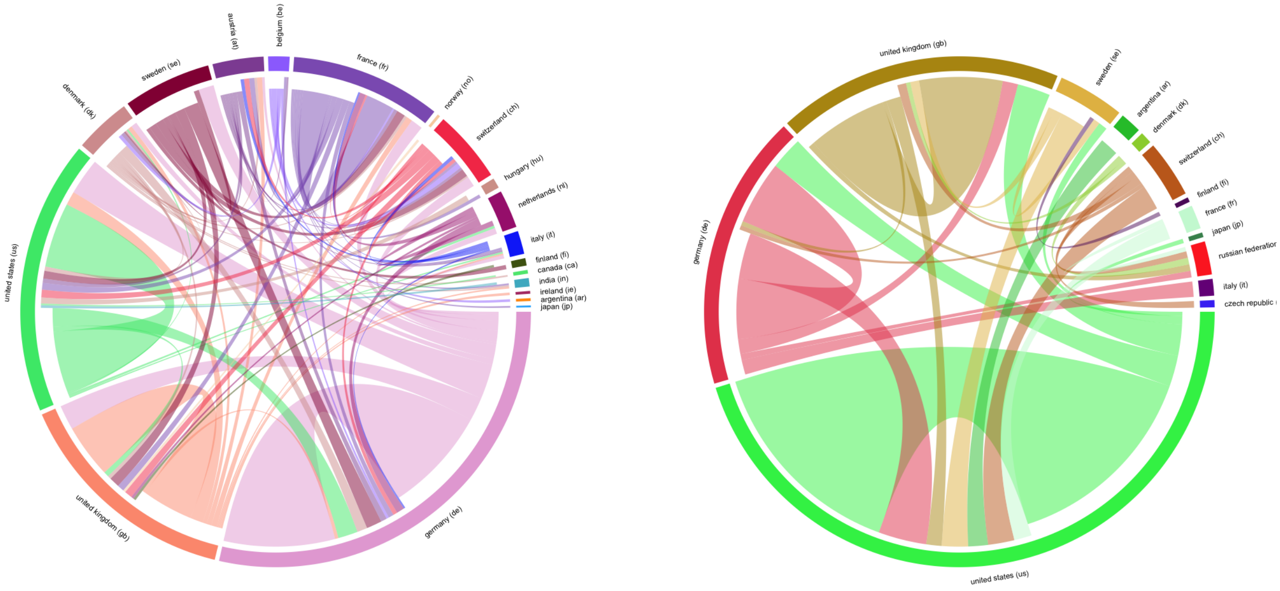

Supplementary Figure 3. Nomination networks at country level. The volume of nominee-nominator relationships, aggregated at country level, before (left) and after (right) WWII. The rise of USA and the fall of Germany is evident, despite the data of the latter period covers 20 years, against the almost 40 years covered by the former period.

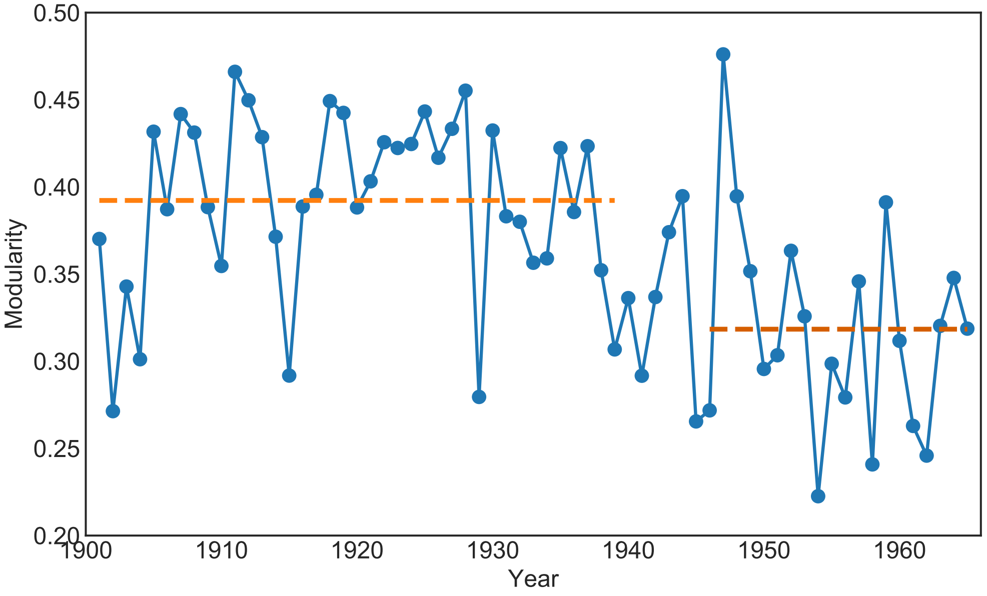

Supplementary Figure 4. Country modularity year-by-year. We observe an evolution in the modularity of the nominator-nominee network. The dashed lines represent the averages for the intervals [1901,1939] and [1946,1965]. The value of country modularity drops from≈ 0.39 before WWII to ≈ 0.32 after the end of the conflict.

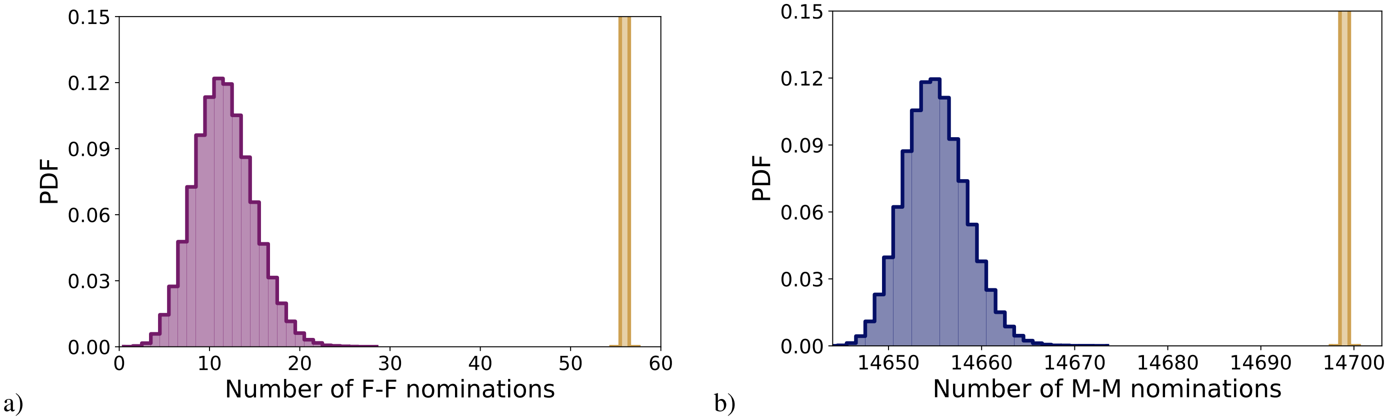

Supplementary Figure 5. Gender homophily – null model The null model is replicated 100000 times. The distribution produced is homogeneous and far from the experimental data (golden line) for both the $F \rightarrow F$ case (panel a) and the $M \rightarrow M$ (panel b). In both cases, nominators nominate more often candidate of the same gender than would be expected if gender would not matter.

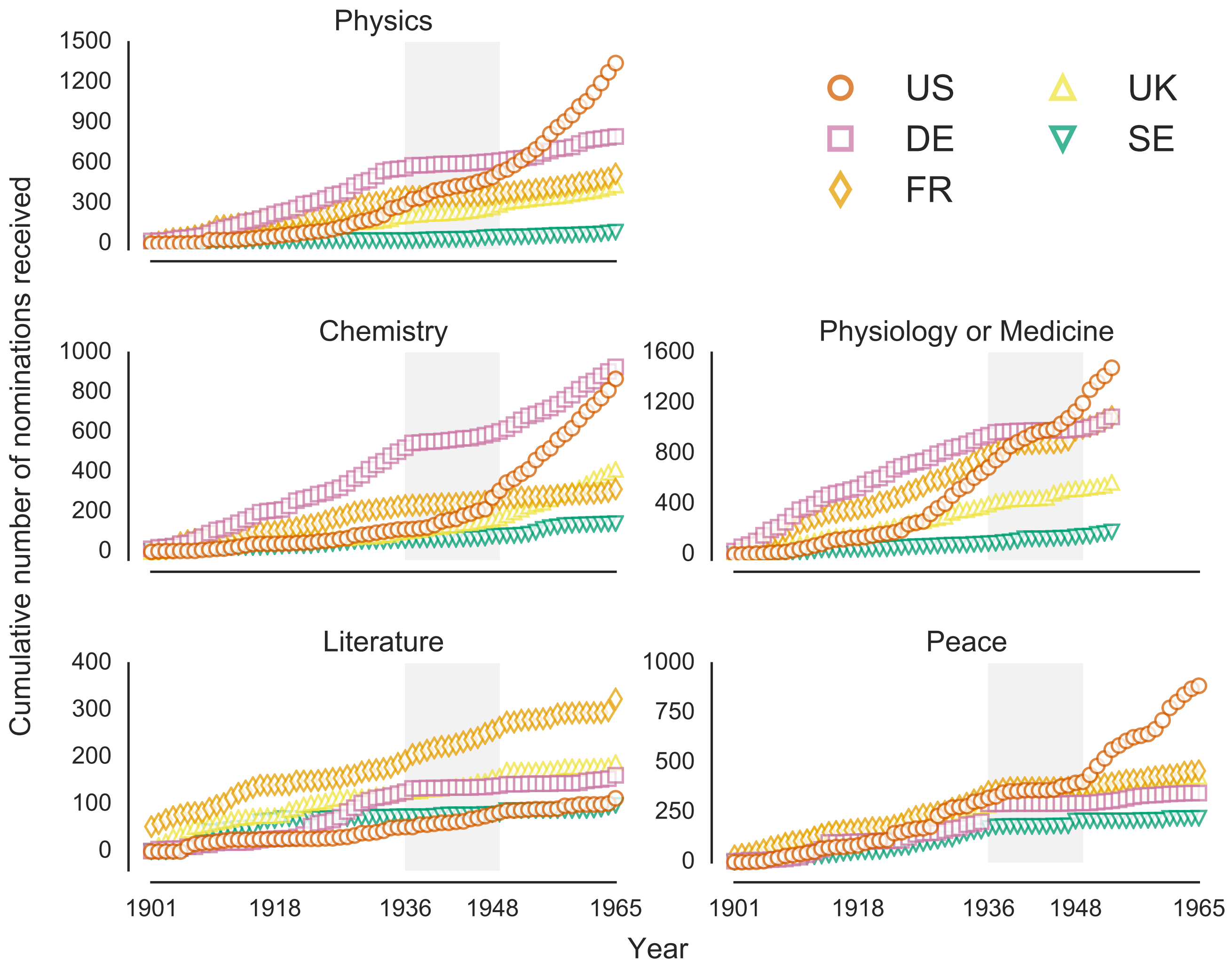

Supplementary Figure 6. **The aggregated number of candidatures casted until a given year for a nominee of a given country.** Comparing with Fig. 3, we see how many of the patterns observed among the nominators are reflected in the candidatures. One notable exception is the predominance of French candidatures in the Prize for Literature, although Fig. 3 highlights the predominance of Swedish nominators.

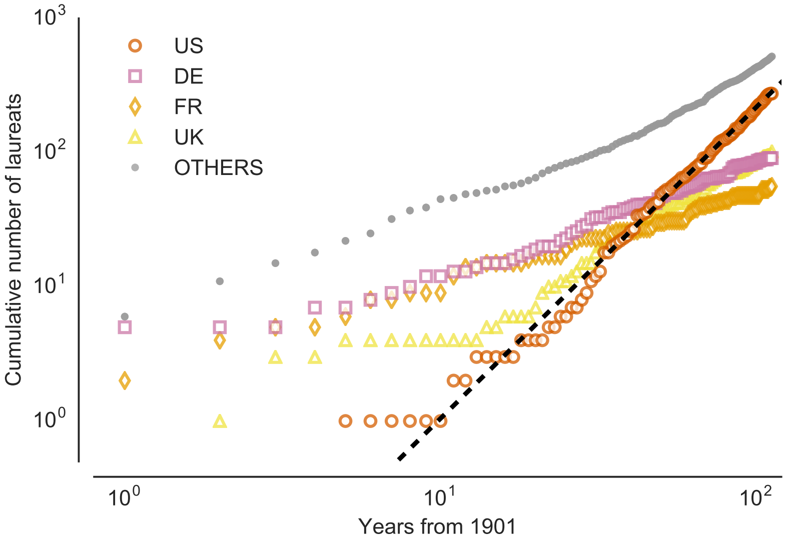

Supplementary Figure 7. The aggregated number of laureates until a given year for the four most awarded countries. The same data of Fig. 2 right, displayed in logarithmic scales and extended until 2016, illustrate how the USA follow a super-linear growth dynamics with a scaling exponent $\gamma \approx 2.3$ (dashed line). An exponent $\gamma = 2$ corresponds to a linear growth of the winning probability, while a $\gamma = 1$ corresponds to a constant winning probability. The latter is observed for Germany, United Kingdom and France.

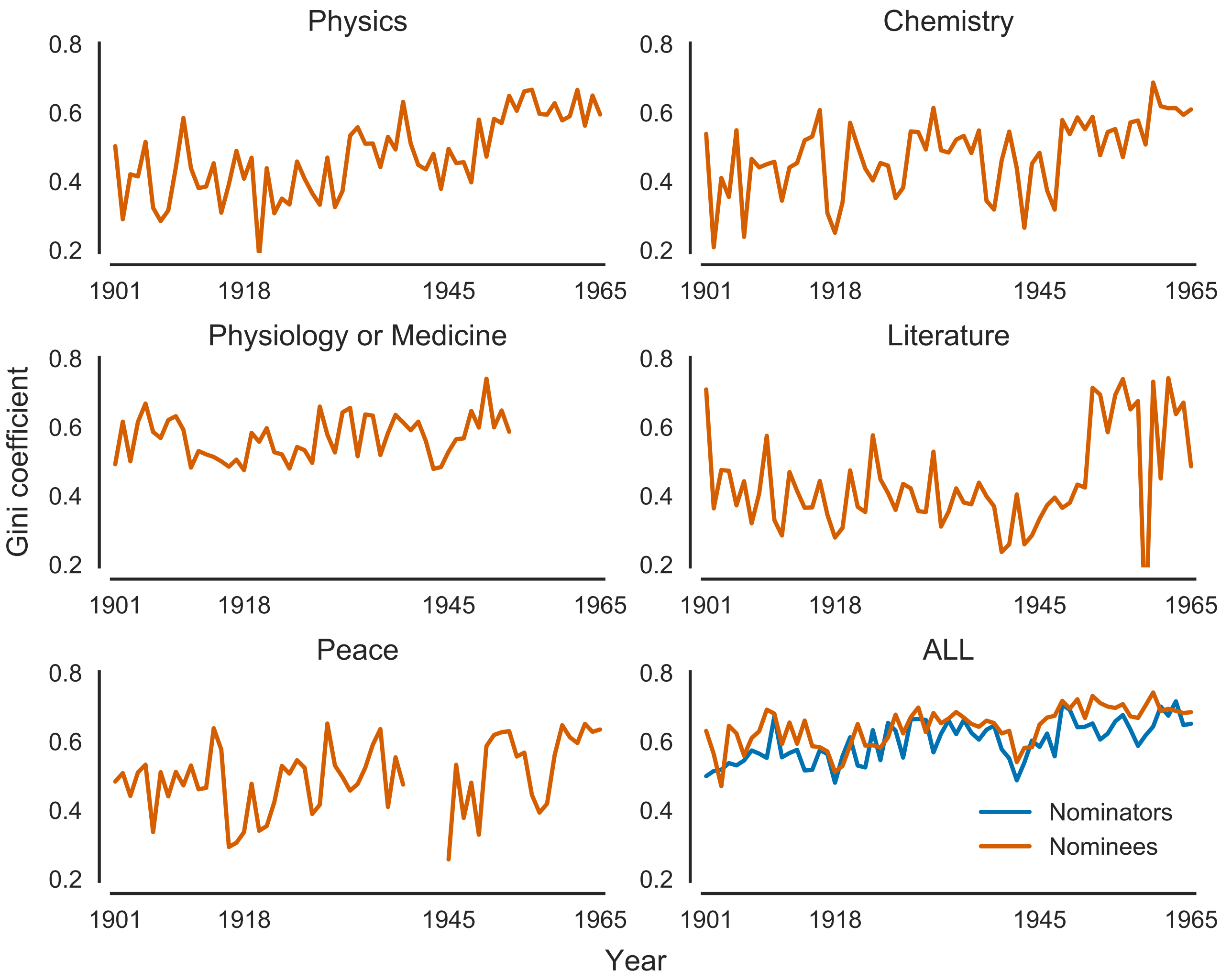

Supplementary Figure 8. Candidatures concentration for the different categories. The growing trend of the Gini coefficient represented in Fig.4c (here replicated in the bottom-right panel) can be observed for all categories.

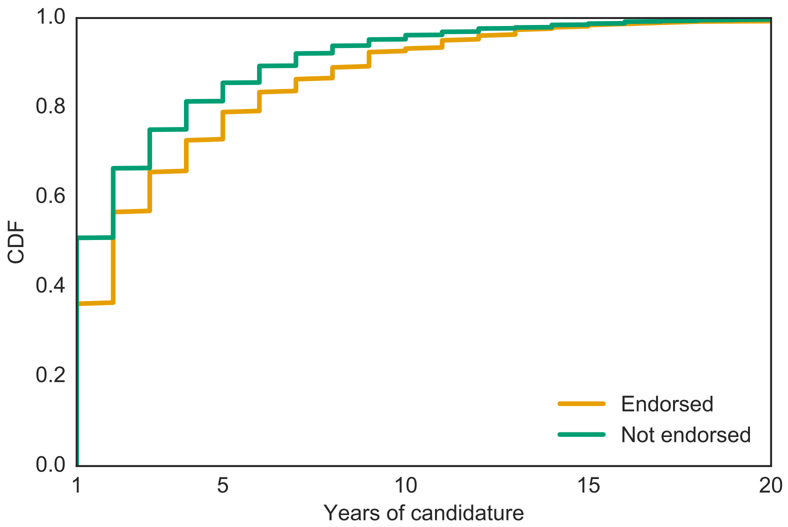

Supplementary Figure 9. Years of candidature for endorsed and not-endorsed nominees. Comparing the two Cumulative Density Functions (CDF) for the number of different years the nominee receive at least a nomination, we remark that endorsed nominees (orange) stay in the loop of the nomination process longer than not-endorsed nominees (green).

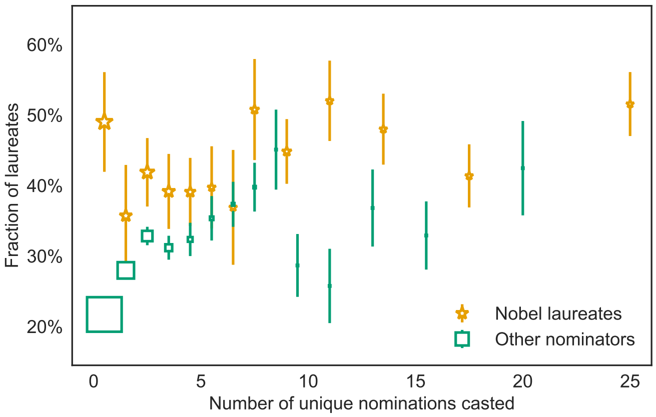

Supplementary Figure 10. Superinfluencers. Nodes with the largest out-degree, a measure that quantifies the most influential nominators, tend to determine more who will be the future Nobel laureates. We measure this by plotting the out-degree against the fraction of nominations to a nominee that will then receive a Nobel prize. Every datapoint averages the win-rates of nominations of at least 10 different nominators. Similarly to Fig. 5a,b, if the nominator is also a Nobel laureate, the probability of having the nominee awarded with a nobel Prize is more than doubled. With the notable exception of single nominations from a former nobel laureate, we observe a growing trend, suggesting the existence of a group of super-influencers that cast numerous “effective” nominations.

Non-overlapping communities: geometric effects in a discrete D-dimensional feature space

The feature space can be continuous or discrete. In the continuous case, every element f_j of the feature vector $\vec{F}$ is a real number in between -1 and $+1$. In the discrete case, only the values -1 and $+1$ are allowed. For a discrete feature space, the possible values of the similarity S are quantised. For instance, in 2 dimensions, vectors $\vec{F}$ can be of four different groups: $(+1,+1)$, $(+1,-1)$, $(-1,+1)$, and $(-1,-1)$. Vectors are therefore either identical, opposite, or orthogonal. The $cossim(\vec{F}_1,\vec{F}_2)$ can assume values -1 if opposite, 0 if orthogonal, and 1 if identical. Follows that $S_2 \in \{0, 0.5, 1\}$. More in general in D-dimensions:

$$S_D \in \{0, 1/D, 2/D, \dots, (D-1)/D, 1\}\,.$$

The number n_d of couple of vectors with similarity $S_D = d/D$, with $d \in 0,1,\dots,D$ is given by the binomial coefficients $n_d = \binom{D}{k}$. Being n_d distributed following a binomial, i the features are distributed homogeneously, most of the nodes will be at intermediate values of similarity.

This discretisation allows us to compute the limiting values of the network modularity for $H_T \to 1$ (that is, a highly selective process). Indeed, as said above, the link creation is dominated by the inequality $Bs_{i_2} + (1-B)S(\vec{F}_1,\vec{F}_2) \geq H_T$. For how the quantities s and S are designed, for $H_T > 1$ no connection is possible. Moreover, being $s \leq 1$ we have

$$H_T > B + (1-B)(1-1/D) = H_T^*(B,D)\,,$$

so all identical nodes are connected between them, while all different nodes are disconnected, no matter the value of s.

The fact that all identical nodes form fully connected cliques allow us to compute the network modularity Q^* expected for $H_T > H_T^*(B,D)$. The modularity is defined as the fraction of edges within the group (here $= 1$) minus the number expected value for a random rewiring. The total number of groups depends on the dimensionality $n_{groups} = 2^D$ (where the value 2 comes from the assumption that the feature space is limited to two options $\{-1,1\}$). Therefore the limiting modularity $Q^*(D)$ for a homogeneous feature distribution is $Q^*(D) = 1 - 1/n_{groups} = 1 - 2^{-D}$.

In Fig. 6c in the manuscript we show the numerical simulation for dimensions ranging between 1 and 4 for $B = 0.2$. For all dimensions D, we can observe how the more the process is selective (growing H_T), the higher the modularity up to the value H_T^* (dash line) where the limiting value Q^* is reached (line-dot lines).

Naturally, also how much the selection is influenced by the meritocracy B, that controls how much the selection is based on the individual score s. The combined effect of selectivity H_T and meritocracy B is illustrated in Supplementary Figure 11, where again we assume the features to be distributed evenly among the population.

For $B = 1$, links are formed only with nodes whose score exceeds the threshold $s_i > H_T$, independently of their features. As a consequence, the modularity is zero ($Q = 0$, the system is feature-blind), the expected number of edges in the system is $\langle E \rangle = L(1-H_T)N$ and the average score of a connected node is $\langle s \rangle = (1+H_T)/2$. In general we expect therefore that the higher H_T, the more selective the process is, the less connections are made and towards better nodes.

This is not true in the opposite extreme $B = 0$, where only the modularity matters. For $H_T > H_T^*$ all and only the 2^{-D} nodes with $S = 1$ are connected with links, so we have $\langle E \rangle = 1/2^D LN$ and an averages score of $\langle s \rangle = 0.5$ because every node with $S = 1$ is connected independently by its merit (the process is merit-blind). Decreasing H_T, the value of $\langle s \rangle$ does not change if we lower the threshold, because we only introduce groups of users with similarity $S_D = d/D$, with $d \in 0,1,\dots,D$, whenever $H_T = d/D$, independently by the score, while the number of links grows accordingly with increments given by $\Delta\langle E \rangle_d = \binom{D}{k}/2^D LN$.

It is also relevant to notice in Supplementary Fig. 11 that in correspondence of the D transitions at $H_T = d/D$ for $d \in 0,1,\dots,D-1$, representing the levels of H_T above which a group of farther similarity gets excluded for $B = 0$, we have that the process become efficiently selective (low E/N, high $\langle s \rangle$) already for low values of B. To illustrate this, consider the case $D = 2$ and $B = 0.1$ for H_T around the value 0.5:

- $H_T = 0.5$, includes 100% of the nodes $S = 1$ plus the best 50% of the nodes $S = 0.5$ for an average score of $\langle s \rangle = 0.625$;
- $H_T = 0.6$, includes 100% of the nodes $S = 1, \langle s \rangle = 0.5$;
- $H_T = 0.4$, includes 100% of the nodes $S = 1$ and 100% of the nodes $S = 0.5$, $\langle s \rangle = 0.5$.

Therefore, even if the system the selection is largely dominated by the homophily, for H_T next to similarity gaps the process is still capable of selecting above average nodes since only the best nodes of the further categories are selected. In this window of values, the effect shown in Fig. 6d in the manuscript appears. The amplitude of the window of values of H_T for this selection grows as B increases.

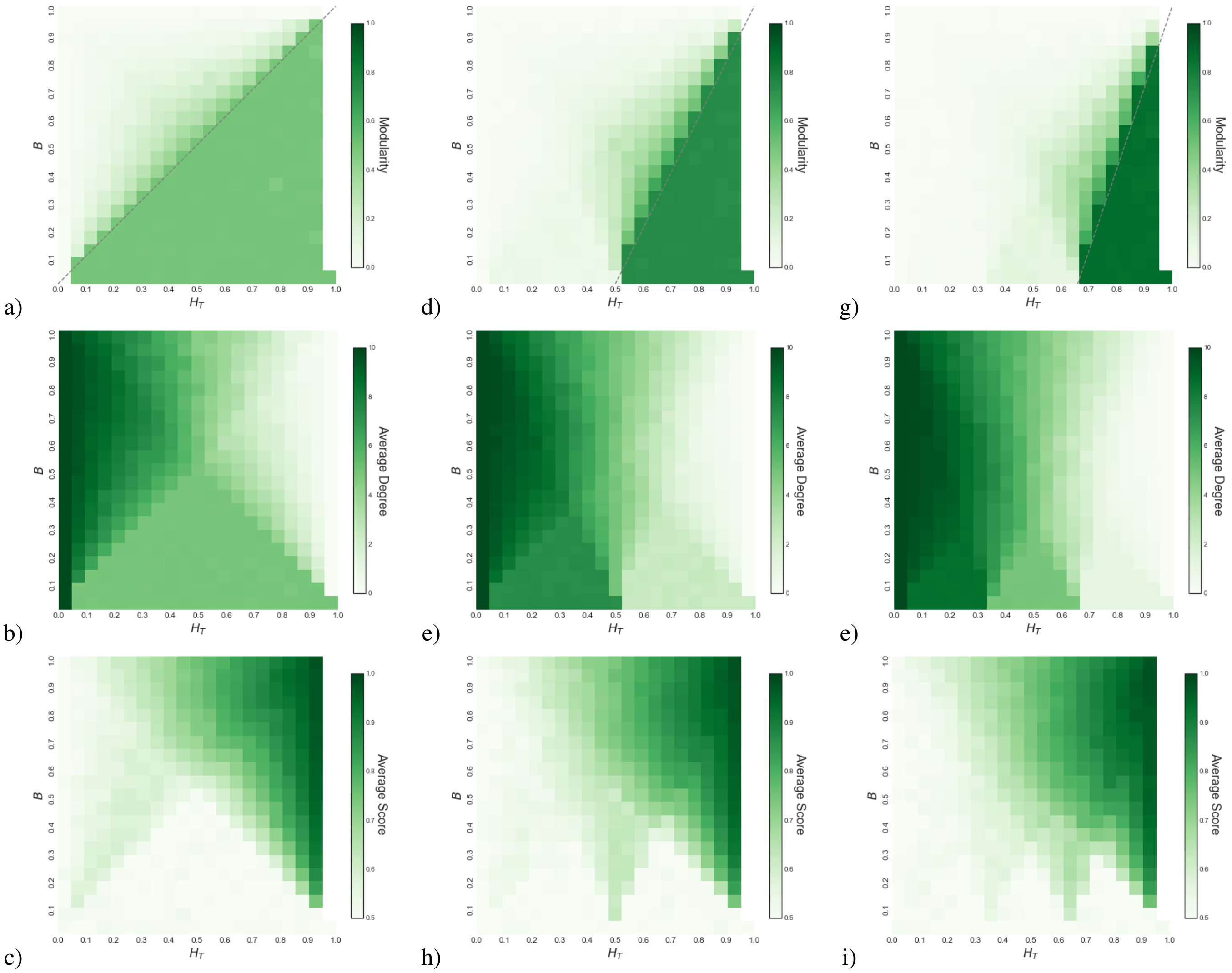

Supplementary Figure 11. How modularity, average degree and average score depends on B, H_T in 1,2,3-dimensional cubic features. Here we present the result of numerical simulations for random features uniformly distributed in the discrete feature space, random values of s uniformly distributed in $[0,1]$, $D = 2$ (left) and $D = 3$ (right), $N_0 = 15$, $N = 1000$, $L = 10$, $\alpha = 0$ (no ageing), $M = 0$ (no special influence). Below the diagonal dashed line (panels a-d-g), described by $H_T = B + (1-B)(1-1/D)$, we have the fully segregated regime where nominations are cast only between nodes of identical features. When the average degree is low, the high process selects nodes of higher score. This is possible even for relatively low values of the meritocracy B as a consequence of the effect illustrated in Fig. 6d in the manuscript.

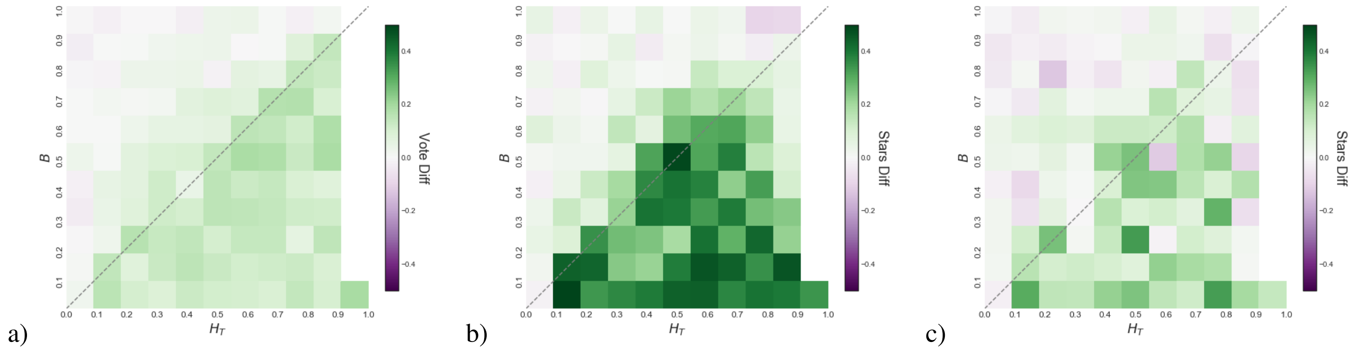

Supplementary Figure 12. Dependence of the effect of gender hegemony on the free parameters B and H_T. Here we measure the gender unbalance as the cumulative relative difference in the number of awards assigned among the two genders after a long time ($100T$ time-steps, where $T = 10$ is the periodicity of the award),. In this figure, we decided to ignore the initial transient phase of $M \cdot T$ time steps required to eventually have a buffer of laureates to use as nominators and focus on the following part, and we average over 20 iterations for every scenario and parameter couple. We study here only three of the four different scenarios illustrated in in Fig. 7b. In panel **a)** we have the baseline scenario without aging and where we do not add the laureates to the nominators pool. Here the gender hegemony is present, but mild. In panel **b)** we introduce the laureates as new nominators and the gender unbalance present is much higher, showing that because of this feature, the network has the emergent property of sustaining and consolidating hegemonic initial conditions. Lastly, in panel **c)** we show that introducing a negative aging exponent ($\alpha = -3$ in the example) can compensate for the gender unbalance induced by the Laureate nominators: if the nominator pool is equally made by new young nodes and former laureates, the effect due to hegemonic initial condition and high homophily is limited.

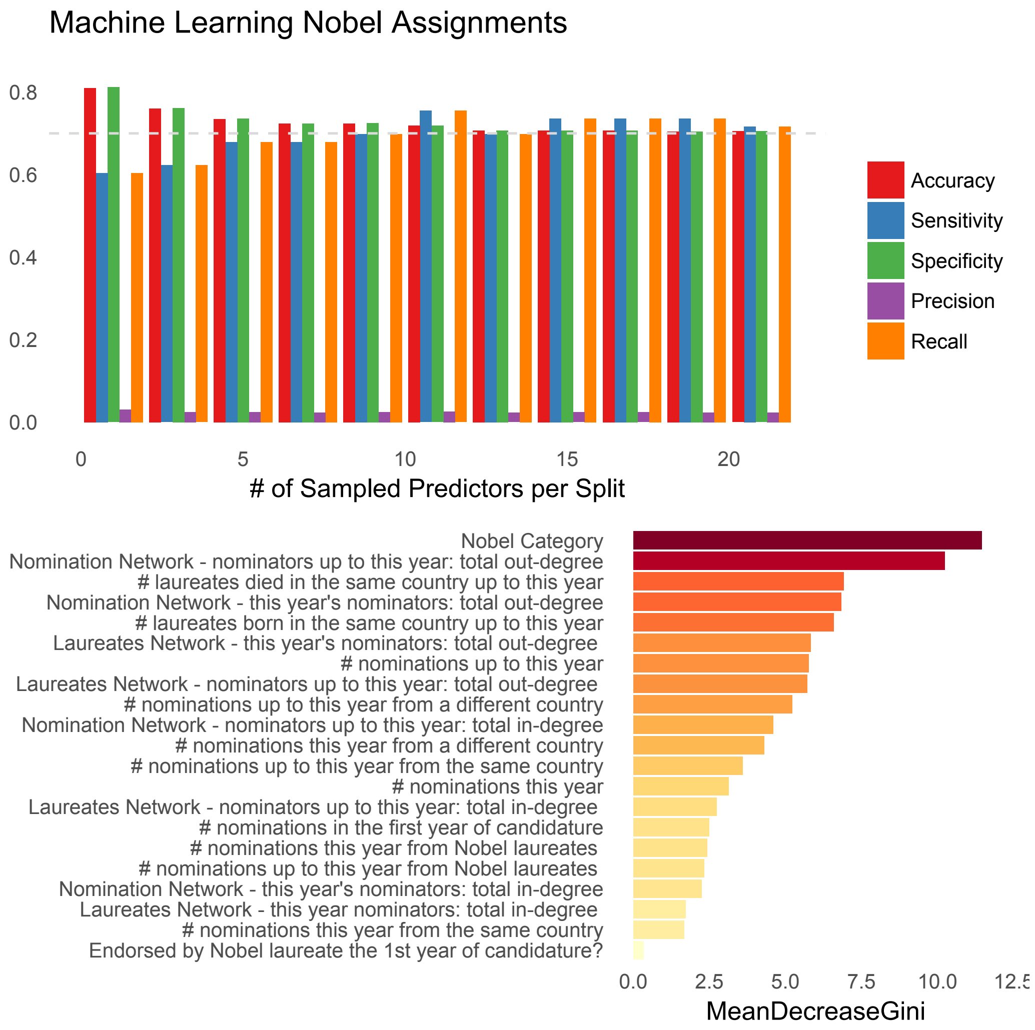

Supplementary Figure 13. Machine learning the Nobel assignment. We feed a machine learning algorithm (specifically, Random Forest) with descriptors and metadata describing the state of the nomination system, with the ultimate goal of predicting the Nobel Prize assignment. The results show that, knowing the history of the nomination-nominee network, it is possible to predict who will win the Nobel Prize with high accuracy ($> 70\%$), as shown in the top panel, in a robust way with respect to the parameters of the algorithm. The bottom panel shows the importance of network features and metadata – as measured from the Mean Decrease in Gini index – used for the training: the Nobel category is a highly predictive feature, together with the number of nominations casted cumulatively (out-degree), the cumulative number of nominations received, as well as the fact that those nominations come from a former Nobel laureate, especially from the same country of the nominee.
